# The Effect of Sound and Stimulus Expectation on Transcranial Magnetic Stimulation-Elicited Motor Evoked Potentials

**DOI:** 10.1007/s10548-021-00867-9

**Published:** 2021-09-06

**Authors:** Antonio Capozio, Samit Chakrabarty, Sarah Astill

**Affiliations:** School of Biomedical Sciences, Faculty of Biological Sciences, University of Leeds, Leeds, UK

**Keywords:** TMS, Discharging noise, Stimulus expectation, Inter-pulse interval, Corticospinal excitability

## Abstract

The amplitude of motor-evoked potentials (MEPs) elicited by transcranial magnetic stimulation (TMS) over the motor cortex is influenced by multiple factors. TMS delivery is accompanied by an abrupt clicking noise which can induce a startle response. This study investigated how masking/attenuating the sound produced by the TMS system discharging influences MEP amplitudes. In addition, the effects of increasing the time between consecutive stimuli and of making participants aware of the time at which they would be stimulated were studied. MEPs were recorded from the Flexor Carpi Radialis (FCR) muscle at rest by stimulation at motor threshold (MT), 120% MT and 140% MT intensity. Participants (N = 23) received stimulation under normal (NORMAL) conditions and while: wearing sound-attenuating earmuffs (EAR); listening to white noise (NOISE); the interval between stimuli were prolonged (LONG); stimulation timing was presented on a screen (READY). The results showed that masking (*p* = 0.020) and attenuating (*p* = 0.004) the incoming sound significantly reduced the amplitude of MEPs recorded across the intensities of stimulation. Increasing the interval between pulses had no effect on the recorded traces if a jitter was introduced (*p* = 1), but making participants aware of stimulation timing decreased MEP amplitudes (*p* = 0.049). These findings suggest that the sound produced by TMS at discharging increases MEP amplitudes and that MEP amplitudes are influenced by stimulus expectation. These confounding factors need to be considered when using TMS to assess corticospinal excitability.

## Introduction

TMS is a non-invasive technique that can be used to study changes in the excitability of the motor system in both experimental (Pascual-Leone et al. 1994) and clinical settings (Hamzei et al. 2006). A single TMS pulse, when applied to the primary motor cortex (M1), can elicit an MEP in the muscles induced by descending activity along the corticospinal tract, as measured via electromyography (EMG) (Hallett 2007). The amplitude of the MEP is suggested to reflect excitability and integrity of local neural networks and their corticospinal projections (Merton and Morton 1980). However, part of the descending activity constituting the MEP is conveyed through indirect (e.g. disynaptic and polysynaptic) cortical and subcortical circuits and is thereby impossible to study the corticospinal component in isolation via EMG recording (Burke and Pierrot-Deseilligny 2010). Multiple sensory and psychological factors can influence the effects of TMS delivered to the motor cortex (Duecker and Sack 2015), limiting the validity of the results in terms of corticospinal excitability.

The discharging of a TMS coil is accompanied by an abrupt clicking noise which increases with stimulation intensity and can reach 120 dB (Nikouline et al. 1999). Neuroimaging data show that magnetic stimulation, even when given at small intensities, induces bilateral activation in the auditory cortex (Bestmann et al. 2004). The auditory activation correlates with the amplitude of the delivered TMS pulse (Goetz et al. 2015). Moreover, auditory stimuli might activate the reticulospinal tract and therefore modulate the excitability of spinal motoneurons (Dean and Baker 2017), which ultimately determines the outcome of TMS on the motor cortex (Burke and Pierrot-Deseilligny 2010). Fisher and colleagues recorded responses from ponto-medullary reticular formation (PMRF) neurons in primates after TMS delivery (Fisher et al. 2012). They found that M1 stimulation produced responses in these neurons which are independent from the descending activity induced by the magnetic field, since the same neurons could be similarly activated by a click stimulus. This class of neurons have mono and disynaptic excitatory projections to spinal motor neurons, and their activation by sound can potentially affect the amplitude of the MEP recorded from the muscle of interest. These data suggest that if MEPs induced by TMS are being used to assess activity in the corticospinal tract, caution is warranted as activation in other cortical and subcortical structures is common, and the MEP may not only be a result of excitability of the motor cortex. Some of the methods employed to mitigate the effects of the TMS clicking noise, widely used when recording electroencephalographic (EEG) signals alongside TMS delivery, include using earplugs and playing white noise through earphones (Fuggetta et al. 2005; Julkunen et al. 2008). The use of earplugs for hearing protection is also recommended by *The Safety of TMS Consensus Group* guidelines (Rossi et al. 2009). Nevertheless, the neuromodulating effect of stimulation noise when TMS is delivered on the motor cortex remains unknown (Goetz et al. 2015) and no studies to date have investigated the effects of masking and attenuating the discharging noise on the recorded MEP within the same stimulation pulse.

Another factor which strongly influences the outcome of TMS is the excitability of the motor cortex at the time of stimulation, a factor known as state dependency (Siebner et al. 2009). In the motor system, cortical excitability is modulated by the phase of the ongoing neural oscillation (Thut et al. 2017). MEP amplitudes recorded from hand muscles are bigger if a TMS pulse is delivered at the troughs and rising edges of the sensorimotor μ-alpha rhythm (Bergmann et al. 2019). In addition, cortical excitability is modulated by action preparation (Rossini et al. 1988) and can be manipulated by asking participants to perform behavioural tasks prior or at the time of stimulation (Mars et al. 2007). For example, in the context of a reaction-time task in which participants respond to a cue with a specified movement, MEP amplitudes recorded from muscles involved in the movement increase before the movement (Chen and Hallett 1999). Moreover, corticospinal excitability is closely correlated with expectancy, increasing when the probability of the response stimulus, instructing to move, to occur at a certain time is higher (van Elswijk et al. 2007). These findings are relevant for conditions in which a motor response has to be exhibited, but might not apply to conditions in which the participant is “at rest” (Tran et al. 2021). Tran and colleagues (2021) addressed this issue by designing a condition in which participants passively attended to a clock on the screen indicating when they were going to receive TMS. In the majority of trials, TMS was delivered on time with the clock (On time-condition). In a small percentage of trials, however, the TMS pulse was instead delivered before (Early-condition) or after (Late-condition) what the clock suggested. These conditions were compared to a baseline condition in which TMS was delivered when participants watched a blank screen. The authors found that stimulus expectation decreased motor excitability, since MEPs recorded in the on time and Late conditions were smaller to the baseline ones (Tran et al. 2021). However, responses were recorded in the context of visual attentional tasks, while MEPs are often recorded with participants not attending to any stimuli (Rossini et al. 1994). Even in the absence of visual stimuli, TMS is accompanied by characteristic auditory and somatosensory stimuli (Nikouline et al. 1999), and participant might build temporal expectation about these events (De Lange et al. 2018). In the context of TMS studies, the temporal relationship between subsequent pulses is dictated by the inter-pulse interval (IPI) (Vaseghi et al. 2015). Longer IPIs (10–15 s) have been shown to induce bigger MEPs compared to short IPIs (5 s) (Hassanzahraee et al. 2019), a phenomenon attributed to the drop in haemoglobin levels, which in turn reduced neural activation, lasting up to 8–10 s after stimulation (Thomson et al. 2012). Habituation to acoustic stimuli has been observed at inter-trial intervals of 5 s, but not at longer intervals (Furubayashi et al. 2000). Nevertheless, to our knowledge the possibility that temporal prediction of the TMS stimulus modulates corticospinal excitability was never investigated.

Given the above, the present study had two clear aims: (1) to investigate the effect of the attenuation or masking of the sound made by the TMS system at discharge on the amplitude of MEPs; (2) to determine whether we can prevent stimulus expectation by increasing and “jittering” the IPI, and whether this phenomenon could be reversed by explicitly making the participants aware of the timing when they would receive the next stimulus. With respect to aim 1, we expected to record significantly lower MEP amplitude values in the conditions reducing or masking the discharging sound compared to the condition where participants received stimulation without sound reduction/masking. This expectation is based on previous studies showing a reduction of the auditory activation induced by TMS when using earplugs and white noise (Ter Braack et al. 2015). Our second hypothesis (aim 2) is that MEPs obtained when using long IPIs would be higher compared to a condition in which the IPI is shorter, since habituation to acoustic stimuli decreases with increasing IPIs (Furubayashi et al. 2000; Nivison et al. 1987). However, we predicted that this effect could be reversed when participants were aware of the time of stimulation, which would indicate that stimulus expectation reduced MEP amplitudes.

## Materials and Methods

### Participants

A total of 23 healthy participants (M ± SD = 22.6 ± 4.2; F = 10) volunteered for the study. Inclusion criteria included being right-handed, since asymmetry between the left and right hands have been observed when delivering TMS (Triggs et al. 1999), and aged between 18 and 40 years. Participants were excluded from the study if they had familiar history of epilepsy or neurological disorders, were under any medication affecting the CNS, or had any contraindications to TMS (Rossi et al. 2009). All participants gave written informed consent and the experimental procedures were approved by the Faculty of Biological Sciences Ethical Review Committee at the University of Leeds and conformed to the Declaration of Helsinki.

### Electromyographic (EMG) Measures

Participants were tested while sitting on a dynamometer chair (Biodex Corp., Shirley, NY), with the right forearm in full pronation, the elbow and the head both fully supported. We recorded electromyography (EMG) activity from the right FCR muscle using a parallel-bar wireless sensor (3.7 × 2.6 cm) (Trigno, Delsys Inc., Natick, MA, USA). Raw EMG recordings were pre-amplified (gain = 909), recorded with a 20–450 Hz bandwidth and digitized at 2 kHz using data acquisition software (Spike2, Cambridge electronics Design, Cambridge, UK).

### Stimulation Technique

Magnetic stimulation was applied to the left motor area M1 by means of a Magstim Rapid stimulator and a flat alpha coil (D70 Alpha Flat Coil, Magstim Company, Whitland, Dyfed, UK) being held by a support stand (Magstim AFC Support Stand, Magstim Company, Whitland, Dyfed, UK). The coil was oriented at ∼45°, inducing a posterior-to-anterior current flow across the motor cortex, and moved across the left motor cortex while delivering stimulation in order to locate the optimal coil position to stimulate FCR (Rossini et al. 2015). The position was marked with a non-permanent marker to ensure consistency of recordings over the session. The positions and orientations of the coil was monitored continuously, and if necessary, adjusted to align with the scalp markings. During all the interventions, the stimulation was controlled through Spike2 (Cambridge Electronic Design, Cambridge, UK) software. We estimated for each subject an individual resting MT, the smallest intensity of stimulation necessary to elicit peak-to-peak MEP amplitudes of at least 50 μV in at least 5 out of 10 trials with 5 s IPIs, following the relative frequency method (Rossini et al. 1994). MT values were used to calculate the intensities to be set during the recording phase.

### Experimental Design

**Fig. 1:**
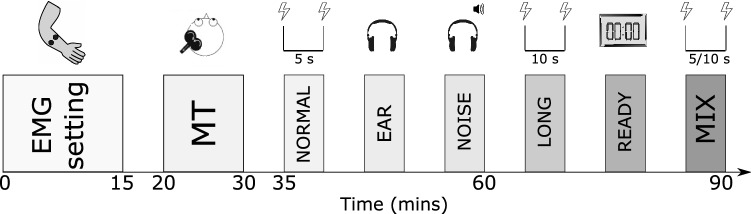
Time course of the experimental session. After electrode placement, an individual MT was estimated for each participant. The experimental conditions (see Methods for details) were then delivered in a randomised order, spaced by 5 min. Each experimental condition lasted between 3 and 5 min and the total duration of each session was approximately 90 min

Common to all experimental conditions, we recorded MEPs by delivering TMS at three intensities: 100% of MT; 120% of MT; 140% of MT. We recorded 10 traces at each of the three intensities of stimulation. The order of delivery was randomized across conditions and participants. All participants were unaware of the rationale of the study and the nature of each experimental condition. Neither the experimenter nor the participant could see the amplitude of the elicited MEPs at the time of stimulation. A total of six different stimulation conditions (outlined below) were completed and the respective MEPs recorded for each participant (Fig. 1). The order of conditions was randomized for each participant.

#### NORMAL Condition

This condition was designed to mimic protocols commonly used to measure the excitability of the corticospinal tract. Participants were asked to relax throughout the stimulation, maintaining their eyes open but without paying attention to any visual cue. The IPI between successive stimuli varied between 4 and 6 s (20% jitter). A total of 30 (3 intensities × 10 traces) MEPs were collected during this phase. The total duration of the sequence was approximately 3 mins.

#### EAR Condition

For this condition, participants were provided with sound-attenuating earmuffs (Peltor Optime, III, 3 M, Maplewood, U.S.) to wear throughout the stimulation protocol. Wearing these attenuates the incoming “click” sound by an average 35 dB across all frequencies (single number rating). This condition was implemented to test whether the intensity of the noise produced by TMS delivery influenced the EMG response to stimulation of the motor cortex. The IPI was again jittered between 4 and 6 s, for a total session length of approximately 3 mins (10 traces × 3 stimulation intensities).

#### NOISE Condition

Participants were asked to wear closed-back headphones through which white noise (frequency range 20–20,000 Hz) at 83 dB of intensity as measured through sound level meter was played while stimulating M1 and recording MEPs. The amplitude was chosen to mask the sound produced by stimulation given at 60% of maximum stimulator output (MSO) (Dhamne et al. 2014). This was confirmed by stimulating the motor cortex at 60% MSO and asking participants to report if they could perceive the sound produced by the magnetic pulse. The position of the headband on the scalp was adjusted such that it didn’t interfere with the coil to ensure consistent coil positioning across conditions. Ten traces (IPIs between 4 and 6 s) for each of the three stimulation intensities were recorded during this phase, lasting approximately 3 mins.

#### LONG Condition

This condition was designed to estimate the effects of increasing the IPI on the recorded MEPs. The IPI between successive stimuli varied between 8 and 12 s (20% jitter), since acoustic stimulus habituation is not observed at longer (> 5 s) IPIs (Furubayashi et al. 2000). A total of 30 (3 intensities × 10 traces) MEPs were collected during this phase. The total duration of the sequence was approximately 6 mins.

#### READY Condition

The same parameters used for the LONG condition were employed for the READY condition: IPIs varying between 8 and 12 s (20% jitter) and a total of 30 (3 intensities × 10 traces) collected responses. However, participants received visual feedback in the form of a stopwatch informing them on the time when they would receive the next stimulus (countdown to 00:00). The countdown to the next stimulus was showed through the TMS stimulator graphical interface. This feature was designed to prevent occurrence of the startling effect that a TMS pulse might induce when delivered unexpectedly. The total duration of the sequence was approximately 6 mins.

#### MIX Condition

In order to further assess whether the length of the IPI influences the amplitude of the recorded responses, we included a condition in which long (8–12 s) and short (4–6 s) IPSs were randomly intermixed. We recorded 5 responses for each combination of IPIs and stimulation intensity (100_short; 100_long; 120_short; 120_long; 140_short; 140_long) for a total duration of approximately 5 min. The number of recorded MEPs for each intensity and IPI (long/short) was lower to keep the total number of traces of this condition consistent with the other conditions. Only a subset (N = 19) of participants completed this experimental condition.

### Data Analysis

In order to ensure that high levels of baseline noise did not influence the recorded MEPs, single traces were excluded from the analysis whenever the root mean square of the EMG in the 50 ms preceding the MEP onset exceeded 5 μV. Overall, 96.9% (3827/3950) of the traces were retained after this procedure. We calculated the peak-to-peak amplitude for each MEP and averaged the 10 MEPs (or 5 in the MIX condition) recorded for each intensity. Given that TMS amplitude data often reveal skewed distributions and deviations from normality (Nielsen 1996), a natural logarithmic transformation was carried out. A GLM analysis was run using SPSS software (Version 26.0) with an a priori significance level of 0.05. Participant was included as a random factor, with Condition (NORMAL, EAR, NOISE, LONG, READY) and Intensity (100%, 120%, and 140% of MT) included as fixed factors. One outlier was removed to meet the assumption of normality of the distribution of residuals (p = 0.060), but removal did not affect the significance of the results of the GLM analysis. The Levene’s test of equality of error variances showed no violation of the assumption of homogeneity of variance (p = 0.519).

We specified a separate model to estimate the effects of manipulating the IPIs on MEPs amplitudes because: (1) only a subset (N = 19) of participants completed this condition; (2) the number of MEPs collected for each IPI at each intensity was lower in this condition compared to the other conditions (5 vs. 10); (3) the two range of IPIs to be tested (MIX_LONG and MIX_SHORT) were delivered in a random order but during the same condition, as opposed to the other 5 conditions. For the MIX condition, Participant was included as a random factor and IPI (MIX_LONG, MIX_SHORT) derived from the MIX condition and Intensity (100%, 120%, 140% of MT) as fixed factors. No violation of normality of the distribution of residuals could be inferred from the results (p = 0.295). The Levene’s test of equality of error variances showed no violation of the assumption of homogeneity of variance (p = 0.202). Bonferroni corrections were applied to all pairwise comparisons.

## Results

**Fig. 2:**
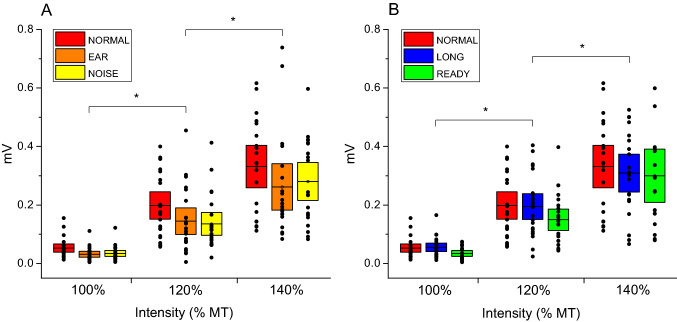
Effects of stimulation intensity on the MEPs amplitudes for different conditions. **A** Individual data and mean (black line) of the MEPs values obtained at 100% (N = 23), 120% (N = 23) and 140% MT (N = 20) intensities for the NORMAL, EAR and NOISE conditions. **B** Individual data and mean (black line) of the MEPs values obtained at 100% (N = 23), 120% (N = 23) and 140% MT (N = 20) intensities for the NORMAL, LONG and READY conditions. The boxes represent the associated 95% confidence intervals. Asterisks denote a significant difference from the lower intensity

**Fig. 3:**
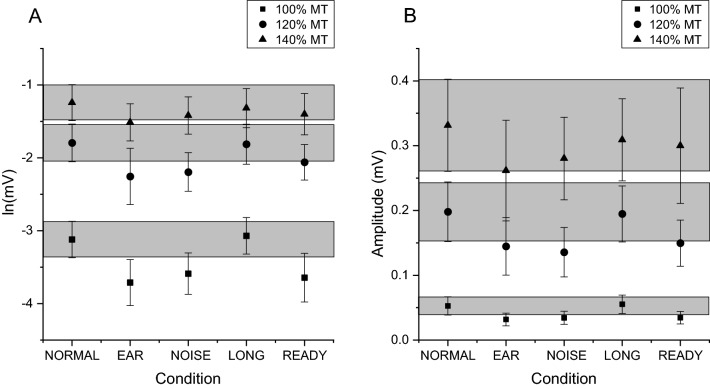
Effects of the experimental conditions on the MEPs amplitudes across different stimulation intensities. **A** Comparison between groups mean natural logarithmic (ln) transformed MEP values (N = 23) obtained across five experimental conditions. **B** Comparison between groups mean raw MEP values (N = 23) obtained across five experimental conditions. The error bars represent the associated 95% confidence intervals

**Fig. 4:**
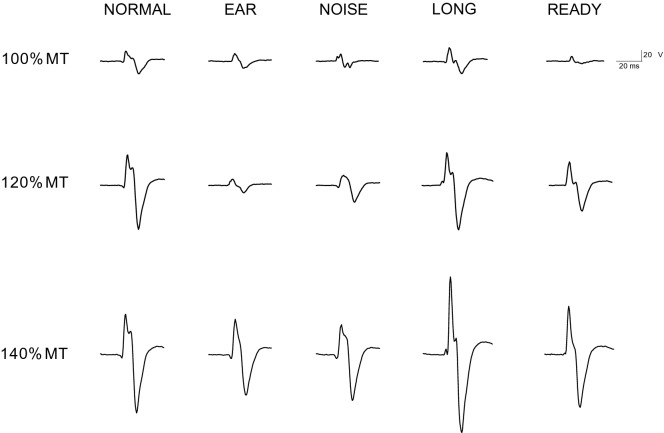
Examples of MEPs evoked by TMS under different conditions and intensities of stimulation in the right FCR muscle of a representative subject. Each trace represents the mean of 10 sweeps

Three participants could not tolerate the 140% MT stimulation intensity and therefore for these three subjects MEPs amplitudes elicited at this intensity were not collected (N = 20). The natural logarithmic transformed amplitude values were used for the GLM analysis. Results from the GLM analysis revealed that the interaction between Intensity and Condition on the amplitude of the MEPs was not significant [F (8, 328) = 0.67, *p* = 0.72, η^2^ = 0.017]. However, we noted a significant main effect of Intensity [*F* (2, 328) = 290.53, *p* < 0.001, η^2^ = 0.649] (Fig. 2), with Bonferroni-corrected post-hoc comparisons (Table 1) showing that MEP values increased from 100% MT to 120% MT intensities (*p* < 0.001) and from 120% MT to 140% MT (*p* < 0.001). There was also a significant effect of Condition [*F* (4, 328) = 6.81, *p* < 0.001, η^2^ = 0.08]. Post-hoc comparisons revealed that MEPs amplitudes were significantly higher in the NORMAL condition compared to the EAR (*p* = 0.002), NOISE (*p* = 0.010) and READY (*p* = 0.041) conditions, but no significant difference was found between NORMAL and LONG condition (*p* = 1) (Fig. 3). Data from a representative participant are displayed in Fig. 4.

**Table 1:** Results of post hoc multiple comparisons

Dependant Variable	Group 1	Group 2	Mean difference	SIG
MEP amplitudes	NORMAL	EAR	0.416	0.002
	NORMAL	NOISE	0.371	0.010
	NORMAL	LONG	− 0.017	1.00
	NORMAL	READY	0.323	0.041

Dependant Variable	Intensity 1	Intensity 2	Mean difference	SIG
MEP amplitudes	100% MT	120% MT	− 1.429	< 0.001
	120% MT	140% MT	− 0.605	< 0.001

**Fig. 5:**
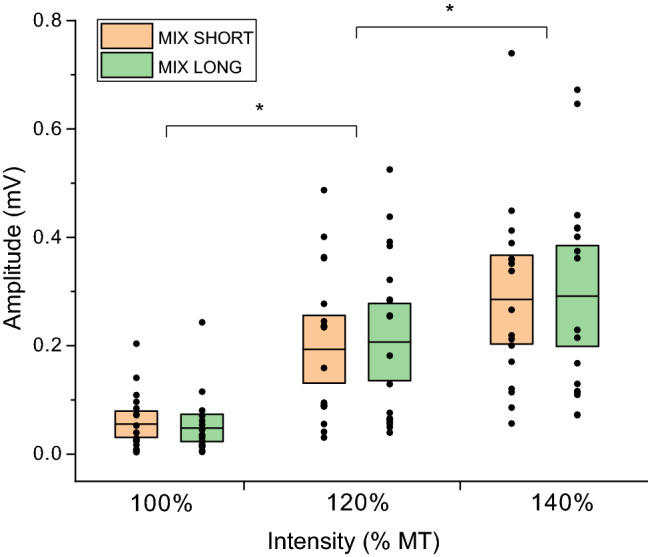
Effects of stimulation intensity on the MEPs amplitudes for different IPIs. Individual data and mean (black line) of the MEPs values obtained at 100% (N = 19), 120% (N = 19) and 140% MT (N = 16) intensities for the MIX SHORT and MIX LONG IPIs. The boxes represent the associated 95% confidence intervals. Asterisks denote a significant difference from the lower intensity

A separate GLM was run to assess the effect of IPI (MIX_SHORT vs MIX_LONG) on MEP amplitudes recorded during the MIX condition (5 traces × 3 intensities × 2 conditions) (Fig. 5). While the main effect of Intensity was significant [*F* (2, 114) = 61.46, *p* < 0.001, η^2^ = 0.532], no significant effect of IPI [*F* (1, 114) = 0.091, *p* = 0.76, η^2^ = 0.001] was observed, and no significant interaction [*F* (2, 114) = 0.038, *p* = 0.96], η^2^ = 0.001].

## Discussion

The main aims of the presented study were to: (1) determine the outcome of attenuating and masking the sound produced by TMS discharging on the MEPs recorded upon M1 stimulation; (2) investigate the effects of stimulus expectation on the MEPs recorded upon M1 stimulation. The data showed that MEP recordings were significantly higher on the NORMAL condition (routinely employed TMS protocol) compared to the EAR (sound attenuating) and NOISE (listening to white noise) conditions. Increasing the IPIs (LONG) had no impact on the MEPs, confirmed by comparing traces recorded with long and short IPIs in the same condition (MIX condition). However, stimulus expectation significantly decreased the activity elicited by TMS in the FCR muscle (READY < NORMAL).

### Attenuating/Masking the Sound

A significant effect of condition showed that MEP amplitudes were lower when using earmuffs compared with the normal condition values. Similarly, MEP values were lower when participants listened to white noise (Fig. 3). Given the nature of the techniques we employed, it is difficult to draw conclusions about the neural populations responsible for the observed effects. Nevertheless, evidence derived from TMS studies on primates (Fisher et al. 2012) and the knowledge of distribution of corticoreticular and reticulospinal axons (Sakai et al. 2009) point to a role of reticular formation neurons in mediating this phenomenon. Reticular formation neurons can be activated by both TMS given on the motor cortex and acoustic stimuli delivered through a bone vibrator (Fisher et al. 2012) and have mono and disynaptic excitatory projections to spinal motor neurons (Baker 2011). We hypothesised that attenuating and masking the incoming sound would lead to a decrease in the number of activated motor neurons at all the intensity of stimulation. In this context, results obtained from the NOISE condition seem paradoxical. Considering that in this condition acoustic stimulation persisted during the whole protocol, we should expect reticular neurons to be repeatedly activated, which in turn would increase spinal excitability (Riddle et al. 2009). However, these neurons show habituation to repeated acoustic stimuli which reduce the synaptic response amplitudes (Yeomans and Frankland 1995). The smaller MEPs we measured in the NOISE condition may thus be explained by habituation to white noise. This finding is partially confounded by the possibility that the headphones used to deliver white noise mechanically attenuated the sound produced by TMS delivery. While not designed for sound-attenuation, the headphones through which white noise was played act as hearing-protection devices and can dampen the incoming sound (Ilmoniemi and Kičić 2010). Another limitation of the current study is that sound travels through bone conduction since the coil is in close contact with the scalp (Nikouline et al. 1999). Adding a layer of foam between the head and the coil has been proved successful in reducing bone conduction (Ter Braack et al. 2015) but this would have affected neural activation too since the induced currents dissipate with distance (O'Shea and Walsh 2007). In addition, even the combined use of noise masking and foam padding cannot completely suppress the auditory and somatosensory perception associated with TMS discharging (Conde et al. 2019). Importantly, however, the EAR and NOISE conditions were not designed to prevent participants’ awareness of stimulation but rather to decrease auditory activation, which would in turn reduce reticulospinal activation (Fisher et al. 2012). Among the limitations of the study, evidence suggests that at least 20 consecutive stimuli should be delivered in order to obtain reliable MEPs recordings (Biabani et al. 2018; Goldsworthy et al. 2016). Further studies should address this issue by recording a higher number of MEPs for each intensity and condition. Nevertheless, excellent inter-session variability was found for measures of corticospinal excitability collected from forearm muscles when only 10 stimuli at each stimulation intensity were employed (Carroll et al. 2001; Carson et al. 2013).

### Stimulus Expectation

In this study the possible influence of stimulus expectation on the recorded MEP amplitudes was also investigated. The data shows no difference between traces obtained using short (5 s, NORMAL) and long (10 s, LONG) IPIs (Fig. 3). These findings are apparently contradictory with the ones reported by Vaseghi and colleagues (Vaseghi et al. 2015), who compared the effect of IPI manipulation on MEP amplitudes (Vaseghi et al. 2015). MEPs given at 10 s IPI were significantly larger than the ones given at 4 s IPI. The authors explained this result in light of the drop in haemoglobin levels, which in turn reduces neural and muscular activation, observed following TMS and lasting up to 8–10 s (Thomson et al. 2012). However, it was observed how MEP amplitudes collected at short intervals (1–3 and 3–5 s) increase over time (Julkunen et al. 2012), which would be inconsistent with this hypothesis. An important methodological difference in our protocol was the introduction of a 20% jitter around the IPI, effectively making the stimulus delivery time harder to predict (Julkunen et al. 2012). We corroborated this finding by designing a condition in which long and short IPIs intermixed, showing no difference between MEPs recorded (Fig. 5). Importantly, for the analysis of the difference between amplitudes recorded at short and long IPIs, only 5 MEPs were collected at each intensity. This number might not be sufficient to derive strong conclusions from our findings (Goldsworthy et al. 2016), and further studies should confirm these results by employing a higher number of stimuli.

Awareness of stimulation time (READY condition) diminished the responses to TMS when compared to the ones recorded with 5 s jittered IPIs (NORMAL condition). Our interpretation of this finding is based on recent findings showing that MEPs collected when stimulation can be anticipated are smaller when compared to these recorded for unexpected stimuli (Tran et al. 2021). Unexpected, loud sounds such as the TMS “click” elicit in mammals a characteristic multisensory response, the acoustic startle response (Davis 1984). The response depends on physiological factors such as fear, attention and habituation (Wassermann et al. 2008). The effect is suppressed when participants are alerted of the stimulation (Hagemann et al. 2006), as in the condition we designed where visual feedback instructed the participants about stimulus delivery. We cannot exclude the possibility that other correlated but independent factors, such as participant’s attention, partially confounded our results. This possibility can be controlled for by designing a condition in which participants are instructed to visualise a cue on the screen without receiving any information about stimulus delivery. Importantly, however, the reduction in MEPs amplitudes observed in the READY condition is unlikely to depend on attentional confounding, since visual attention to external cues was shown to increase the excitability of the motor cortex (Ruge et al. 2014). As previously mentioned, cortical oscillations influence the outcome of TMS on motor cortex (Bergmann et al. 2019; Iscan et al. 2016). MEP amplitudes negatively correlate with increased parietal alpha activity (Zarkowski et al. 2006), and alpha oscillations have been suggested to reflect top-down processing of incoming stimuli (Thut et al. 2017). In the visual system, predictions about incoming visual stimuli increase power in the alpha frequency band (Mayer et al. 2015). Future studies might investigate the potential role of cortical oscillatory activity in mediating our results by measuring EEG during the different conditions we designed. Finally, we monitored the EMG activity in the pre-stimulus phase (up to 50 ms) and discarded traces with high background noise to ensure that no changes in baseline activity (e.g. preactivation) could be observed in any condition. Nevertheless, any subthreshold modulation of corticospinal excitability would go unnoticed by our EMG recordings, and it could be that cortical structures may exert an inhibitory influence on downstream structures and reduce the descending corticospinal volley (Li 2007). Designing a condition in which participants are both anticipating the stimulus arrival and wearing earplugs/headphones to mask the noise will help elucidate the theory that the two effects are not cumulative, but rather mediated by partially overlapping neural pathways.

### Practical Implications for Choosing Experimental Conditions

Given the different nature of the conditions we designed we believe that the choice of a TMS protocol to implement should be based on the specific research question. Use of white noise and earmuffs often pushed MT amplitudes below the value which would be considered threshold by definition (> 50 μV). This issue must be considered when delivering stimulation at increasing percentages of MT value, as was the case in the current study, and interpreting results in terms of corticospinal excitability. Many established protocols are based on the estimation of a MT and on the assumption that subthreshold TMS does not induce descending activity along the corticospinal tract (Kujirai et al. 1993; Nielsen et al. 1993). In these instances, a potential activation of the reticulospinal system by sound should be considered as a confounding factor and therefore controlled for. Therefore, we argue that MT values obtained when using earmuffs might better reflect the activity of corticospinal neurons, without the effects of the acoustic stimulus. To note, use of hearing protection was recommended as a safety measure by *The Safety of TMS Consensus Group* (Rossi et al. 2009), but its potential effects on MEP amplitudes were never investigated. Whether the two conditions we designed (EAR and NOISE) successfully reduced the spread of activity to other pathways needs to be experimentally confirmed, but these constitute interesting alternatives to “classical” TMS protocols. The efficacy of white noise in masking the incoming sound seemed to deteriorate at 140% MT intensities. High stimulation intensities are often employed in diagnostic TMS studies requiring maximal corticomotor response (Rossini et al. 2015). Noise levels need to be adjusted according to the “click” produced to guarantee masking.

While interesting from a theoretical point of view, giving visual feedback to participants to inform of stimulation time is likely to introduce many uncontrollable variables. We instructed our subjects to focus their visual attention on the clock showing delivery time without producing any anticipatory reaction, but the attentional state induced by our instructions depended on individual characteristics and might constitute an additional source of variability. Future studies could potentially address this issue by measuring the activity induced in different neural populations under various stimulation conditions directly in primates and indirectly in humans employing neuroimaging techniques. Methodological information such as instructions to participants and their prior experience of TMS need to be reported even in studies assessing motor excitability at rest. Finally, a further limitation of the current study is that EMG activity was recorded exclusively from the FCR muscle. Further studies should confirm that the decrease in MEP amplitudes recorded while participants wore earphones, listened to noise and were aware of stimulation delivery can be observed over other muscles.

## Conclusions

The present study demonstrated that the sound produced by TMS discharge influences the amount of activity recorded via EMG from the FCR muscle. Masking and attenuating the clicking sound might reduce unintended effects caused by auditory activation and provide a more valid measure of corticospinal excitability to contribute to diagnosis or ascertain efficacy of therapy. Participants’ knowledge of discharging time decreased the amplitude of responses elicited by suprathreshold stimulation at rest. By using a randomized IPI instead of a constant IPI the possible confounding effect of habituation and expectation can be minimized (Schmidt et al. 2009).

## Data Availability

Data supporting the findings of this study are available from the corresponding author, A. C., upon reasonable request.
